# *In vitro* and *in vivo* anti-cancer activities of Kuding tea (*Ilex kudingcha* C.J. Tseng) against oral cancer

**DOI:** 10.3892/etm.2013.1450

**Published:** 2013-12-16

**Authors:** KAI ZHU, GUIJIE LI, PENG SUN, RUI WANG, YU QIAN, XIN ZHAO

**Affiliations:** Department of Biological and Chemical Engineering, Chongqing University of Education, Chongqing 400067, P.R. China

**Keywords:** Kuding tea, buccal mucosa cancer, human tongue carcinoma TCA8113 cells, U14 squamous cell carcinoma cells, metastasis

## Abstract

Kuding tea (*Ilex kudingcha* C.J. Tseng) is drunk widely in China. The *in vitro* anticancer effects of Kuding tea were evaluated in TCA8113 human tongue carcinoma cells using a 3-(4,5-dimethyl-2-thiazolyl)-2,5-diphenyltetrazolium bromide (MTT) assay. At a concentration of 200 μg/ml, Kuding tea exhibited an inhibitory effect of 75% in TCA8113 cells, which was higher than that observed at concentrations of 100 and 50 μg/ml (41 and 10% inhibition, respectively). Reverse transcription-polymerase chain reaction (RT-PCR) and western blot analyses of the apoptosis, inflammation and metastasis genes and proteins in Kuding tea-treated cancer cells were performed. Kuding tea significantly induced apoptosis in TCA8113 cancer cells (P<0.05) by upregulating Bax, caspase-3 and caspase-9 expression, and downregulating Bcl-2 expression. Expression of the NF-κB, iNOS and COX-2 genes that are associated with inflammation was significantly downregulated by Kuding tea, which demonstrated its anti-inflammatory properties. Kuding tea also exerted an anti-metastatic effect on cancer cells. This was demonstrated by the decreased expression of matrix metalloproteases (MMPs) and the increased expression of tissue inhibitors of metalloproteinases (TIMPs), and confirmed by the inhibition of the metastasis of U14 squamous cell carcinoma cells in imprinting control region (ICR) mice. The ICR mouse buccal mucosa cancer model was established by injecting the mice with U14 cells. Following injection, the wound at the injection site was topically treated with Kuding tea. It was observed that the tumor volumes for the group treated with Kuding tea were smaller than those from the control mice. Analysis of the sections of buccal mucosa cancer tissue demonstrated that the buccal mucosa cancer degrees of the Kuding tea-treated mice were weaker than that in the control mice. Similar results were observed in the lesion sections of the cervical lymph nodes. Based on these results, Kuding tea exhibited successful *in vitro* anticancer effects in TCA8113 cells and *in vivo* buccal mucosa cancer preventive activity.

## Introduction

Kuding tea (*Ilex kudingcha* C.J. Tseng) is a beverage consumed in China as an alternative to the more common, green tea (1). Kuding tea has a reputation for preventing deterioration of the heart and brain function and maintaining proper body weight. The main active components are triterpene glycosides (saponins), which have been dubbed kudinosides and kudinlactones; Kuding tea also contains polyphenols and flavonoids, similar to those in ordinary tea (2,3). Caffeoyl quinic acid (CQA) derivatives have also been identified as major phenolic compounds in Kuding tea. CQA derivatives have been isolated from the natural functional compounds of a variety of plants and have demonstrated pharmacological properties in numerous diseases, including as antioxidants, hepatoprotectants, antibacterial agents, antihistamines and as anticancer and neuroprotective agents (4,5).

Oral squamous cell carcinoma is a type of cancer that usually develops in the squamous or epithelial cells that cover the lips and the oral cavity. The malignant or cancerous cells are usually located in the floor of the mouth or on the surface of the tongue (6). Squamous carcinoma cells are located in the epidermis of the skin, and this type of cancer is one of the major forms of skin cancer. Squamous cell carcinoma is the second most common skin cancer (7). The U14 mouse tumor is a squamous cell carcinoma. It is an ectopically-induced carcinoma*,* induced by treating the uterine cervix with 20-methylcholanthrene (8). U14 cells are widely used in studies of tumor invasion, metastasis, recurrence and drug screening. The establishment of a cultured tumor cell line, capable of forming a tumor *in vivo*, is likely to be helpful for the study of tumor biology on a cellular and molecular level (9).

Apoptosis is characterized by a series of typical morphological features, including cell shrinkage, membrane blebbing, chromatin condensation and nuclear fragmentation (10). The induction of apoptosis is a defense against cancer. Apoptosis leads to cell death with cell shrinkage, pyknosis and karyorrhexis. The regulation of cell death may be a significant component in cancer as aberrantly regulated apoptotic cell death causes apoptotic diseases, including cancer. Elucidating the critical events associated with carcinogenesis provides the opportunity to prevent cancer development via dietary intervention by inducing apoptosis, particularly with bioactive agents or functional foods. Drink is a significant environmental factor in the overall cancer process, and is capable of exacerbating or interfering with disease progression. In addition to dietary effects on protein expression and function, there is evidence that a large number of food components may exert effects on the human genome, by directly or indirectly modulating gene expression (11). Balkwill and Mantovani demonstrated that inflammation may predispose an individual to cancer (12). Hallmarks of inflammation-related cancers include the presence of inflammatory cells and mediators in tumor cells. Inflammation may occur in the early stages of cancer, but inflammatory mediators and cells are also involved in the migration, invasion and metastasis of malignant cells (13). Metastasis is the leading cause of mortality among cancer patients and involves the cancer spreading from a primary site and forming new tumors in distant organs. Matrix metalloproteases (MMPs) have an important role in numerous physiological or pathological processes including embryonic development, morphogenesis, reproduction, tissue remodeling, arthritis, cardiovascular disease and metastasis (14). MMP activity is inhibited by specific endogenous tissue inhibitors of metalloproteinases (TIMPs). In order to prevent the majority of cancer types, improved metastasis treatments are required (15).

In the present study, the anticancer and antimetastatic effects of Kuding tea were examined. Kuding tea was administered to TCA8113 human tongue carcinoma cells and the molecular mechanisms underlying the anticancer effects of the tea were studied. The variations in the activities of Kuding tea at different concentrations were evaluated and the antimetastatic effects were assessed in mice with tumors propagated by U14 squamous cell carcinoma cells. An animal model was established in which the mice were injected with U14 cells for the *in vivo* evaluation of buccal mucosa cancer.

## Materials and methods

### *Kuding tea (Ilex kudingcha* C.J. Tseng) *extracts*

Kuding tea was provided by the Henglv Tea Co. (Chongqing, China). The Kuding tea was stored at −80ºC and freeze-dried to produce a powder. The powder was extracted for 24 h with a 20-fold volume of methanol, twice. The methanol extract was evaporated using a rotary evaporator (N-1100; Eyela, Tokyo, Japan), concentrated and subsequently dissolved in DMSO (Amresco; Solon, OH, USA) in order to create the stock concentration (20%, w/v).

### Cancer cells

TCA8113 cells were obtained from the Shanghai Institute of Biochemistry and Cell Biology (SIBCB, Shanghai, China) and the U14 squamous cell carcinoma cells were obtained from the Chinese Academy of Medical Sciences (Beijing, China). The cancer cells were cultured in RPMI-1640 medium (Welgene Inc., Daegu, Korea) supplemented with 10% fetal bovine serum (FBS) and 1% penicillin-streptomycin (Gibco Co., Grand Island, NY, USA) at 37ºC in a humidified atmosphere containing 5% CO_2_ (incubator model 311 S/N29035; Forma, Waltham, MA, USA). The medium was changed two or three times each week (16).

*In vitro* cultured U14 cells (5×10^6^/mouse) were injected into the abdominal cavity of 7-week-old female imprinting control region (ICR) mice. After one week, the carcinoma ascites were obtained and diluted in sterile saline to achieve a concentration of 1×10^7^/ml.

### 3-(4,5-Dimethyl-2-thiazolyl)-2,5-diphenyltetrazolium bromide (MTT) assay

The anticancer effects of Kuding tea were assessed with an MTT assay. The TCA8113 cells were seeded in a 96-well plate (2×10^4^ cells/ml per well) in a volume of 180 μl. Subsequently, 20 μl of the 50, 100 or 200 μg/ml Kuding tea samples were added. The cells were subsequently incubated with the Kuding tea solutions for 48 h at 37ºC in an incubator (model 311 S/N29035) in a humidified atmosphere containing 5% CO_2_. MTT (Amresco) solution (200 μl; 5 mg/ml) was added to each well and the cells were cultured for a further 4 h under the same conditions. Following the removal of the supernatant, 150 μl DMSO was added to each well and mixed for 30 min. Finally, the absorbance of each well was measured using an ELISA plate reader (model 680; Bio-Rad, Hercules, CA, USA) at 540 nm (17).

### Reverse transcription-polymerase chain reaction (RT-PCR) to measure mRNA expression

Total RNA was isolated from TCA8113 cells using TRIzol reagent (Invitrogen, Carlsbad, CA, USA) according to the manufacturer’s instructions. The RNA was digested with RNase (Roche, Basel, Switzerland) for 15 min at 37ºC and purified using an RNeasy kit (Qiagen, Hilden, Germany) according to the manufacturer’s instructions. cDNA was synthesized from 2 μg total RNA by incubating at 37ºC for l h with avian myeloblastosis virus reverse transcriptase (GE Healthcare, Little Chalfont, UK) with random hexanucleotides according to the manufacturer’s instructions. Primer sequences used to specifically amplify the genes of interest are provided in Table I. Amplification was performed in a thermal cycler (T100; Bio-Rad), with cycles at 94ºC for 30 sec, 60ºC for 30 sec, then 72ºC for 30 sec performed 50 times for denaturation. The amplified PCR products were run on 1.0% agarose gels and visualized by ethidium bromide (EtBr) staining (18).

### Protein extraction and western blot analysis

Total cell lysates were obtained with an extraction buffer as described previously (18). Protein concentrations were determined using a protein assay kit (Bio-Rad). For western blot analysis, aliquots of the lysate containing 30–50 μg protein were separated by SDS-PAGE and subsequently electrotransferred to a nitrocellulose membrane (Schleicher and Schuell, Keene, NH, USA). The membranes were subjected to immunoblot analysis and the proteins were visualized using an enhanced chemiluminescence (ECL) assay kit (GE Healthcare). The cell lysates were separated by 12% SDS-PAGE, transferred to a polyvinylidene fluoride membrane (GE Healthcare), blocked with 5% skimmed milk, and incubated with the primary antibodies (1:1,000 dilution). Antibodies against Bax, Bcl-2, caspases, iNOS, COX-2, NF-κB, IκB-α, MMPs and TIMPs were obtained from Santa Cruz Biotechnology, Inc. (Santa Cruz, CA, USA). Following incubation with the horseradish peroxidase-conjugated goat anti-mouse secondary antibodies (Abbkine, Redlands, CA, USA) at room temperature, immunoreactive proteins were detected using a chemiluminescent ECL assay kit (GE Healthcare) according to the manufacturer’s instructions. Bands in the blot were visualized using a LAS3000 luminescent image analyzer (Fujifilm Life Science, Tokyo, Japan).

### Induction of buccal mucosa cancer

Female ICR mice (n=50, 6-weeks-old) were purchased from the Experimental Animal Center of Chongqing Medical University (Chongqing, China). They were maintained in a temperature-controlled (temperature 25±2ºC, relative humidity 50±5%) facility with a 12-hour light/dark cycle and had unlimited access to a standard mouse chow diet and water.

To investigate the preventive effect of the Kuding tea against buccal mucosa cancer induced by injecting U14 cells into the mice, the mice were divided into five groups with ten mice in each group. The experiment design was as follows: Kuding tea solutions (Group A: 400 mg/kg, Group B: 800 mg/kg, Group C: 1,600 mg/kg) were administered to the three groups, respectively, by gavage, and Kuding tea solutions (Group A: 100 mg/ml, Group B: 200 mg/ml, Group C: 400 mg/ml) were topically applied to the buccal mucosa of the mice in the three Kuding tea groups once every 12 h for 14 days. The control and Kuding tea groups were subsequently inoculated with the U14 cancer cell suspension (1×10^7^/ml; 0.05 ml per mouse) on the buccal mucosa. The normal group mice received no treatment during the experiment. The mice were sacrificed 14 days later and their tumor volumes and lymph node metastasis rates were determined (16).

### Histological grading of buccal mucosa cancer

Buccal mucosa and lymph node tissues were removed and embedded in paraffin for histological analysis with hematoxylin and eosin staining. The buccal mucosa cancers were graded as follows: I, well-differentiated carcinoma, cells appear much like the adjacent benign squamous epithelium; II, moderately differentiated carcinoma, cells form large anastomosing areas in which keratin pearls are formed; they are not numerous and the main component consists of cells with pronounced cytonuclear atypia; III, poorly differentiated carcinoma: cells have lost the majority of their squamous epithelial characteristics and architecture (16).

### Statistical analysis

Analysis of variance (ANOVA) was performed and the results are presented as the means ± SD. Differences between mean values of the individual groups were assessed with a one-way ANOVA and Duncan’s multiple range tests. P<0.05 was considered to indicate a statistically significant difference. The SAS v9.1 statistical software package (SAS Institute Inc., Cary, NC, USA) was used to perform all statistical analyses.

## Results

### Kuding tea induced the inhibition of TCA8113 cell growth

The inhibitory growth effect of Kuding tea on TCA8113 cells was evaluated using an MTT assay. When the Kuding tea methanol extracts were added to the TCA8113 cells, the growth inhibition rates associated with the 50, 100 and 200 μg/ml extracts were 10, 41 and 75%, respectively (P<0.05, Table II). These results demonstrated that Kuding tea has an antiproliferative effect on TCA8113 cells.

### Apoptosis-related expression of Bax, Bcl-2 and caspases

**Figure 1:**
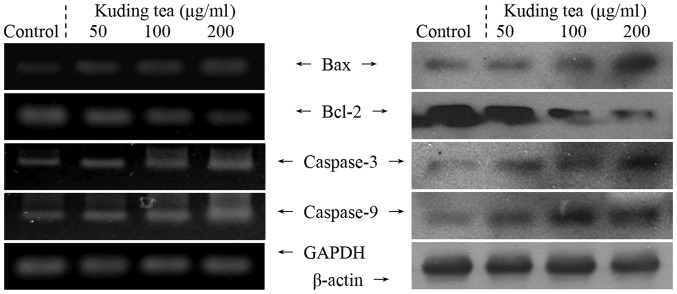
Effects of various concentrations of Kuding tea on the mRNA (left) and protein (right) expression levels of Bax, Bcl-2, caspase-3 and caspase-9 in human tongue carcinoma TCA8113 cells.

The expression levels of Bax, Bcl-2, and caspase-3 and caspase-9 mRNA and proteins in TCA8113 cells were analyzed by RT-PCR and western blotting, respectively, following 48 h incubation with 50, 100 and 200 μg/ml solutions of Kuding tea. As shown in Fig. 1, treatment with 200 μg/ml Kuding tea markedly altered the levels of pro-apoptotic Bax and anti-apoptotic Bcl-2. Bax mRNA and protein expression levels were upregulated, while those of Bcl-2 were decreased significantly. These results suggest that a high concentration of Kuding tea markedly induced apoptosis in TCA8113 cells via a Bcl-2-dependent pathway compared with a low concentration. Modest mRNA and protein expression levels of caspase-9 and -3 were detected in the untreated control TCA8113 cells, but higher expression levels were detected once the cells had been treated with the Kuding tea samples. Notably, the mRNA expression levels of caspase-3 and -9 significantly increased in the presence of the Kuding tea with increasing extract concentrations.

### Inflammation-related expression of NF-κB, IκB-α, iNOS and COX-2

**Figure 2:**
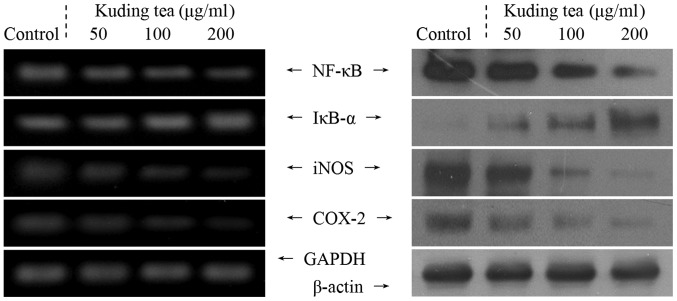
Effects of various concentrations of Kuding tea on the mRNA (left) and protein (right) expression levels of NF-κB, IκB-α, iNOS and COX-2 in human tongue carcinoma TCA8113 cells.

Subsequently, the correlation of the anticancer characteristics of Kuding tea with the inhibition of the expression of the inflammation-related genes NF-κB, IκB-α, iNOS, and COX-2 was investigated. At a concentration of 200 μg/ml, Kuding tea demonstrated anti-inflammatory activity in the TCA8113 cells, with decreased mRNA and protein expression of NF-κB along with increased IκB-α expression compared with that in cells treated with the other concentrations of Kuding tea (Fig. 2). As shown in Fig. 2, the levels of mRNA and protein expression of COX-2 and iNOS were high in the untreated control TCA8113 cells; however, these indicators were almost undetectable in the presence of 200 μg/ml Kuding tea. Following incubation with 200 μg/ml Kuding tea, the mRNA and protein levels of COX-2 and iNOS were significantly decreased.

### Metastasis-related MMP and TIMP expression

**Figure 3:**
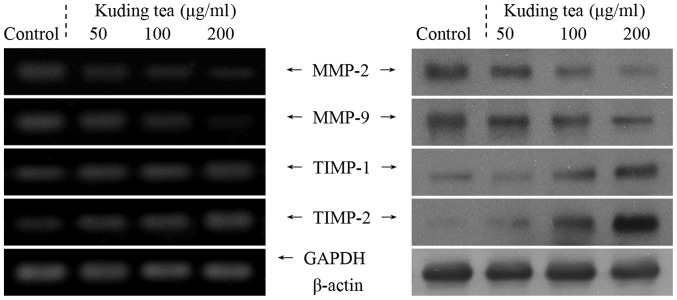
Effects of various concentrations of Kuding tea on the mRNA (left) and protein (right) expression levels of MMPs and TIMPs in human tongue carcinoma TCA8113 cells.

RT-PCR and western blot analyses were performed in order to determine whether the antimetastatic effect of the Kuding tea was due to the genetic regulation of metastatic mediators, specifically MMPs (MMP-2 and MMP-9) and TIMPs (TIMP-1 and TIMP-2), in TCA8113 cells. As shown in Fig. 3, Kuding tea significantly decreased the mRNA and protein expression levels of MMP-2 and MMP-9 and increased the expression levels of TIMP-1 and TIMP-2. These changes in TIMP and MMP expression resulting from Kuding tea treatment may lead to metastatic inhibition *in vitro*. The results also demonstrated that Kuding tea had strong antimetastatic activity.

### In vivo preventive effects on buccal mucosa cancer of Kuding tea

Buccal mucosa cancer was induced by injecting U14 cells into the mice. After 14 days, the mice in all of the groups exhibited carcinogenesis. The tumor volumes of buccal mucosa tissues were measured. The tumor volumes for the control and A, B and C Kuding tea groups were 12.4, 11.8, 9.6 and 6.4 mm^3^, respectively (Table III). Five mice in the control group, five in Group A, four in Group B and two in Group C exhibited lymph node metastasis. Consequently, the lymph node metastasis rates were 50, 50, 40 and 20%, respectively. These results demonstrated that Kuding tea was effective in impeding carcinogenesis, proliferation and metastasis.

**Figure 4:**
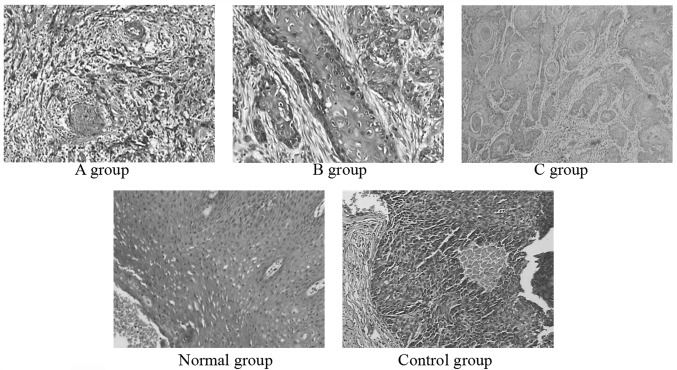
Histology of buccal mucosa tissues induced by injecting U14 squamous cell carcinoma cells into mice (H&E staining; magnification, ×100). Group A, mice were administered 400 mg/kg by gavage and topically treated with 100 mg/ml Kunming tea; Group B, mice were administered 800 mg/kg by gavage and topically treated with 200 mg/ml Kunming tea; Group C, mice were administered 1,600 mg/kg by gavage and topically treated with 400 mg/ml Kunming tea.

Histological changes in the buccal mucosa of the mice injected with U14 cells were examined by hematoxylin and eosin staining. The histological tissue sections of mice in the normal group exhibited the normal histological morphology of squamous epithelium tissue. Histopathological evaluation revealed indications of buccal mucosa cancer in all groups receiving U14 cells (Fig. 4). The sections from the mice in Group A and the control group demonstrated that all tissue had lost its squamous epithelial characteristics and architecture, but the tissues from Group A demonstrated intracellular bridging between the normal squamous cells. The histopathological sections indicated that mice in Group A and the control group developed poorly differentiated carcinoma (grade III), and the control group experienced more serious carcinogenesis. The tumor cells of the mice in Group B were in nests, and there were certain larger, eosinophilic, polygonal cells that were attempting to layer themselves in a squamous-like fashion. However, for Group B, the overall resemblance to a normal squamous epithelium was less clear (grade II). The tissue sections of Group C appeared less like normal squamous epithelium. The cells appeared similar to those of the adjacent benign squamous epithelium (grade I). From these sections, Kuding tea demonstrated a preventive effect on buccal mucosa cancer.

## Discussion

Although Kuding tea is a traditional drink, little scientific data on its effects are available. *Ilex kudingcha* C.J. Tseng, also known as Kuding tea, has high levels of ursolic acid, β-amyrin, lupeol and taraxerol (19). Kuding tea has been reported to have various therapeutic effects, including hepatoprotective, antibacterial, antihistamine and anticancer activities, on numerous pathologic conditions (4). Kuding tea has also been shown to exhibit *in vitro* anticancer effects on human nasopharyngeal carcinoma cells according to the MTT assay (20).

The induction of apoptosis in cancer cells is a promising approach for cancer therapy (21). In the normal cell, the anti-apoptotic Bcl-2 gene is expressed on the outer mitochondrial membrane surface (22). As the Bax and Bcl-2 genes are mostly expressed during apoptosis, it has been determined that these genes regulate apoptotic activity. Apoptosis results from the activation of caspases that act as aspartate-specific proteases (23). The results of the present study demonstrated that changes in Bax and Bcl-2 expression correlated with apoptosis promoted by the Kuding tea. Apoptosis results from the activation of caspase family members that act as aspartate-specific proteases. Caspases form a proteolytic network within the cell whereby upstream initiator caspases are activated early in the apoptotic process (caspase-9) and in turn activate other downstream caspases (caspase-3). Downstream of the region in which apoptosis is initiated in the mitochondrial pathway, caspase-9 and caspase-3 play major roles. Caspase-3 appears to amplify caspase-9 initiation signals to induce a fully fledged commitment to nuclear disassembly (24,25). NF-κB is present in the cytosol where it is bound to the inhibitory protein, IκB. Following its induction by a variety of agents, NF-κB is released from IκB and translocates to the nucleus where it binds to the κB binding sites in the promoter regions of the target genes (27). NF-κB is involved in the inhibition of apoptosis, stimulation of cell proliferation, inflammation, immune response and tumorigenesis. The activation of NF-κB normally prevents apoptosis. iNOS and COX-2, two genes regulated by NF-κB, which are induced by inflammation, are frequently overexpressed in cancer cells. Increased NF-κB activity localized in the nucleus is particularly identified within cells where there is abundant expression of iNOS and COX-2 (28). It has been suggested that COX-2 has an important role in colon carcinogenesis, and NOS, in addition to iNOS, may be a successful target for cancer chemoprevention (26). These mechanisms may be involved in the anticancer effects of Kuding tea on cancer cells. Based on the results of the MTT assay and the expression patterns of pro-apoptotic genes observed in the present study, it was concluded that cancer cells treated with Kuding tea underwent apoptosis.

MMPs, a family of zinc-dependent endopeptidases, play an important role in tumorigenesis and metastasis. MMPs are capable of cleaving almost all extracellular matrix (ECM) substrates. Degradation of the ECM is a key event in tumor progression, invasion and metastasis (29). Among the MMP family members, MMP-2 and MMP-9 are molecules crucial for cancer invasion (30), and are highly expressed in breast and colon cancer cells (31). Notably, the inhibition of MMP activity is useful for controlling tumorigenesis and metastasis (32). TIMPs are naturally occurring inhibitors of MMPs that prevent catalytic activity by binding to activated MMPs, thereby blocking ECM breakdown (33). Disturbances in the ratio between MMPs and TIMPs have been observed during tumorigenesis (34). Maintaining the balance between MMPs and TIMPs or increasing TIMP activity are useful methods for controlling tumor metastasis (35). Experimental evidence demonstrating the role of MMPs in metastasis has been obtained by *in vitro* invasion assays (36).

MMP-2 and MMP-9 are key factors in cancer cell invasion and metastasis *in vitro* (37). Spontaneous and experimental metastasis to the liver decreased in mice that overexpressed TIMP1, and increased in mice that expressed antisense TIMP-1 mRNA. The ectopic overexpression of TIMP-1 in the brains of transgenic mice also reduced experimental metastasis to the brain (38). Notably, MMP-2 and MMP-9 are important for tumor invasion and angiogenesis. Thus, tumor metastasis may be inhibited by blocking MMP synthesis and activity (39).

Histopathology is an important tool in anatomical pathology, as the accurate diagnosis of cancer usually requires the histopathological examination of samples. Histopathology is an important clinical standard for diagnosing oral cancer (40). As buccal mucosa cancer is the most common cancer of the oral cavity (41), the cancer-preventive effect of Kuding tea was evaluated and confirmed using the buccal mucosa cancer mouse model. Accordingly, Kuding tea may be expected to contribute to the prevention of buccal mucosa cancer.

In conclusion, various *in vitro* experimental methods, including MTT, RT-PCR, and western blotting assays, were used to evaluate the anticancer effects of Kuding tea. The *in vivo* anticancer effects of Kuding tea were confirmed in mice injected with U14 squamous cell carcinoma cells. The results from the present study demonstrated that the *in vivo* antimetastatic and anticancer effects of high concentrations of Kuding tea were stronger than those of low concentrations. Overall, Kuding tea demonstrated potent *in vitro* and *in vivo* anticancer activities. The active compounds present in Kuding tea require further identification and evaluation.

## Figures and Tables

**Table I:** Sequences of RT-PCR primers used in this study.

Gene name	Sequence
Bax	Forward: 5′-AAG CTG AGC GAG TGT CTC CGG CG-3 Reverse: 5′-CAG ATG CCG GTT CAG GTA CTC AGT C-3′
Bcl-2	Forward: 5′-CTC GTC GCT ACC GTC GTG ACT TGG-3′ Reverse: 5′-CAG ATG CCG GTT CAG GTA CTC AGT C-3′
Caspase-9	Forward: 5′-GGC CCT TCC TCG CTT CAT CTC-3′ Reverse: 5′-GGT CCT TGG GCC TTC CTG GTA T-3′
Caspase-3	Forward: 5′-CAA ACT TTT TCA GAG GGG ATC G-3′ Reverse: 5′-GCA TAC TGT TTC AGC ATG GCA-3′
NF-κB	Forward: 5′-CAC TTA TGG ACA ACT ATG AGG TCT CTG G-3′ Reverse: 5′-CTG TCT TGT GGA CAA CGC AGT GGA ATT TTA GG-3′
IκB-α	Forward: 5′-GCT GAA GAA GGA GCG GCT ACT-3′ Reverse: 5′-TCG TAC TCC TCG TCT TTC ATG GA-3′
iNOS	Forward: 5′-AGA GAG ATC GGG TTC ACA-3′ Reverse: 5′-CAC AGA ACT GAG GGT ACA-3′
COX-2	Forward: 5′-TTA AAA TGA GAT TGT CCG AA-3′ Reverse: 5′-AGA TCA CCT CTG CCT GAG TA-3′
MMP-2	Forward: 5′-CTT CTT CAA GGA CCG GTT CA-3′ Reverse: 5′-GCT GGC TGA GTA CCA GTA-3′
MMP-9	Forward: 5′-TGG GCT ACG TGA CCT ATG AC-3′ Reverse: 5′-GCC CAG CCC ACC TCC ACT CC-3′
TIMP-1	Forward: 5′-GTC AGT GAG AAG CAA GTC GA-3′ Reverse: 5′-ATG TTC TTC TCT GTG ACC CA-3′
TIMP-2	Forward: 5′-TGG GGA CAC CAG AAG TCA AC-3′ Reverse: 5′-TTT TCA GAG CCT TGG AGG AG-3′
GAPDH	Forward: 5′-CGG AGT CAA CGG ATT TGG TC-3′ Reverse: 5′-AGC CTT CTC CAT GGT CGT GA-3′

RT-PCR, reverse transcription-polymerase chain reaction.

**Table II:** Growth inhibition of TCA8113 human tongue carcinoma cells by various concentrations of Kuding tea as evaluated by an MTT assay.

	OD_540_		
	
Treatment	50 μg/ml	100 μg/ml	200 μg/ml
Control		0.497±0.004^a^	
Kuding tea	0.447±0.010^b^ (10)	0.293±0.008^c^ (41)	0.124±0.008^d^ (75)

The values in parentheses are the inhibition rates (%).

a–d Mean values with different letters are considered to be significantly different (P<0.05) according to Duncan’s multiple range test.

MTT, 3-(4,5-dimethylthiazol-2-yl)-2,5-diphenyltetrazolium bromide; OD, optical density.

**Table III:** Tumor volumes and lymph node metastasis rates of mice topically treated with various concentrations of Kuding tea.

Group	Normal	Control	Group A	Group B	Group C
Tumor volume (mm^3^)	0	12.4±0.6^a^	11.8±0.8a,b	9.6±0.5^b^	6.4±0.5^c^
Lymph node metastasis rate	0	5/10 (50%)	5/10 (50%)	4/10 (40%)	2/10 (20%)

Metastasis rate is the number of lymph node metastasis / total number of mice.

a–c Mean values with different letters are considered to be significantly different (P<0.05) according to Duncan’s multiple range test. Group A, mice were administered a 400-mg/kg gavage and topically treated with 100 mg/ml Kuding tea; Group B, mice were administered a 800-mg/kg gavage and topically treated with 200 mg/ml Kuding tea; Group C, mice were administered a 1,600-mg/kg gavage and topically treated with 400 mg/ml Kuding tea.
